# Algae-based biofilm productivity utilizing dairy wastewater: effects of temperature and organic carbon concentration

**DOI:** 10.1186/s13036-016-0039-y

**Published:** 2016-12-15

**Authors:** Zachary T. Fica, Ronald C. Sims

**Affiliations:** Utah State University, 4105 Old Main Hill, Logan, UT 84322-4015 USA

**Keywords:** Biofilm, Dairy wastewater, Arrhenius, Temperature correction coefficient, Organic carbon, Nutrient uptake

## Abstract

**Background:**

Biofilm-based microalgal growth was determined as functions of organic chemical loading and water temperature utilizing dairy wastewater from a full-scale dairy farm. The dairy industry is a significant source of wastewater worldwide that could provide an inexpensive and nutrient rich feedstock for the cultivation of algae biomass for use in downstream processing of animal feed and aquaculture applications. Algal biomass was cultivated using a Rotating Algal Biofilm Reactor (RABR) system. The RABR is a biofilm-based technology that has been designed and used to remediate municipal wastewater and was applied to treat dairy wastewater through nutrient uptake, and simultaneously provide biomass for the production of renewable bioproducts.

**Results:**

Aerial algal biofilm growth rates in dairy wastewater at 7 and 27 °C temperatures were shown to be 4.55 ± 0.17 g/m^2^-day and 7.57 ± 1.12 g/m^2^-day ash free dry weight (AFDW), respectively. Analysis of Variance (ANOVA) calculations indicated that both an increase in temperature of the wastewater and an increase in the level of organic carbon, from 300 to 1200 mg L^-1^, contributed significantly to an increase in the rate of biomass growth in the system. However, ANOVA results indicated that the interaction of temperature and organic carbon content was not significantly related to the biofilm-based growth rate.

**Conclusion:**

A microalgae-based biofilm reactor was successfully used to treat turbid dairy wastewater. Temperature and organic carbon concentration had a statistically significant effect on algae-based biofilm productivity and treatment of dairy wastewater. The relationships between temperature, TOC, and productivity developed in this study may be used in the design and assessment of wastewater remediation systems and biomass production systems utilizing algae-based biofilm reactors for treating dairy wastes.

## Background

The Utah State University owned Caine Dairy Teaching and Research Center is among the nation's leading dairy production research centers. Research at the Caine Dairy focuses on animal nutrition and reproduction, waste-handling, animal health, and irrigated pasture for intensive rotational grazing. The center also houses three-hundred head of cattle used for dairy production. The Caine Dairy is equipped with a traditional flush system to clean the feed stations for the cattle, and 2000 gallons of water are used to flush twice daily. The flushed waste is directed through a coarse filter grate and the large solids are removed and dried. The liquid phase is pumped into a one acre settling lagoon, which empties into a one acre evaporation pond, where the wastewater is held until it is pumped to the feed stations to be used for flushing stalls. Recycling used wastewater to flush stalls is a common practice in agriculture [1]. However, recycling water creates a closed system for the liquid waste, where solid waste is the only stream leaving the system. The result is a buildup of water soluble nutrients, such as phosphorus and nitrogen, and turbid wastewater.

One strategy that can be employed with agricultural wastes is land application [2]. However many dairy wastewaters, such as the wastewater at the Caine Dairy, contain high concentrations of nitrogen and phosphorus that are not appropriate for land application, and must undergo costly pretreatment solutions if they are to be land applied [3]. These same compounds can serve as a nutrient source for the production of algal biomass that can be used as a feedstock for downstream processing into bioproducts [4, 5].

The Rotating Algal Biofilm Reactor (RABR) is a biofilm-based reactor system using a partially submerged rotating cylinder with growth substratum attached to the outside of the cylinder and with a novel harvesting mechanism [6]. Biofilm growth is possible even in turbid wastewater systems, because the RABR rotates the biofilm in and out of the water, thereby exposing it to both light and a nutrient source. The RABR provides the possibility for wastewater nutrient sources to be utilized for algae-based systems that could not support suspended algal growth due to turbidity, color, or water depth limitations. Applications of biofilm engineering compared with suspended growth systems offer additional benefits by eliminating the need for polymers, sedimentation, and centrifugation when harvesting [7], and therefore reduce the costs associated with harvesting when compared to traditional suspended growth systems [8]. The harvested biofilms can be used to generate bioproducts through downstream processing including bioplastics, biofuel feedstock, high value pharmaceutical compounds, and nutrient rich animal feed [4, 9–12]. Other biofilm systems, such as the Algal Turf Scrubber, have been investigated as possible algal production strategies; however these biofilm systems are limited by turbidity [5].

Previous studies have been conducted using dairy wastewater as a nutrient source in algae systems [13, 14], and it has been suggested that an algae-based alternative could provide a more cost effective treatment process for dairy and other livestock wastewater sources [15, 16]. In some other algal growth systems, organic carbon has been shown to be antagonistic to phototrophic growth often due to nutrient competition with heterotrophic bacteria [17, 18]. However there is a lack of information in the literature regarding the effect of temperature and organic carbon content on algae growth in dairy wastewater systems. There is also a lack of published information concerning the potential for biofilms to be used to remove nutrients from dairy wastewater and create a renewable source of bioproducts. The objective of this study was to determine the effect of temperature and organic carbon concentration on biofilm biomass productivity and on associated nutrient uptake into algae-based biomass cultivated on dairy wastewater.

## Methods

### Wastewater and culture

Wastewater was collected from the Utah State University Caine Dairy Farm evaporation pond. Characteristics of the water and the produced algae-based biofilm are summarized in Table 1. Turbidity of the water was measured at 890 NTU (Hach 2100Q turbidimeter). Algae biomass inoculum for the laboratory RABRs was collected from the pilot scale RABR systems currently operating at the Logan City wastewater treatment facility, a 460 acre (1.86 km^2^) open lagoons system [19]. Visual microscopy of the pilot scale RABR based biofilm indicated that the collected algae biomass contained a variety of algae species, with *Pseudanabaena*, *Oscillatoria*, and *Chroococcus* as the predominant species. The biomass was then cultivated on a cotton rope substratum in shaker flasks using dairy wastewater as the nutrient source before application to the RABR system to allow the culture to adapt to the nutrient source. The carbon:nitrogen:phosphorus molar ratio of the adapted biofilm was measured to be 85:16:1, which is comparable to other algae-based systems [20].

**Table 1:** Composition of influent Caine Dairy wastewater and cultivated biomass from the RABR system. (Analysis by Chemtech-Ford Laboratories – Sandy, UT)

Chemical composition	Water^a^ (mg L^-1^)	Biomass^b^ (mg kg^-1^ dry wt.)
Total organic carbon	1200	648500
Total nitrogen	155^c^	140400
Total phosphorus	12	19100
Aluminum	7.67	776
Boron	1.73	132
Barium	0.63	75.7
Cobalt	0.03	1.96
Chromium	0.42	45.3
Copper	3.05	85.2
Iron	6.80	624
Manganese	0.67	107
Molybdenum	0.09	27
Sodium	460	23455
Nickel	0.25	24.1
Lead	0.05	6.41
Silica	106	5572
Strontium	1.18	125
Zinc	1.15	116

^a^Dairy wastewater influent stream

^b^Produced algae-based biofilm

^c^Total Kjeldahl Nitrogen (Organic nitrogen, ammonia, and ammonium)

### Growth conditions

**Fig. 1:**
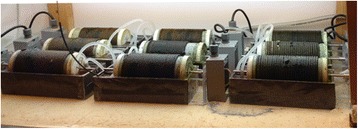
Laboratory scale RABRs. Each set of three RABRs represents a different organic carbon concentration, and all reactors are held at a constant temperature. This experimental design was replicated at three different temperatures, for a total of 27 different reactors. The rectangular base of each RABR contains 1 L of wastewater and the cylindrical portion of the reactors (76 mm diameter, 200 mm length) rotates at approximately 8 rpm. Biofilm accumulates on the cotton rope surface of the cylinder

Rotating Algal Biofilm Reactors of 1-Liter volume were constructed and operated according to Christenson and Sims [6], and the biofilm reactors were wrapped with premeasured lengths of 3/16 in. dia. (0.476 cm dia.) solid braid cotton rope (Fig. 1). In order to test the effect of organic carbon concentration on biomass productivity, the reactors were filled with different dilutions of wastewater and balanced to match the total nitrogen and phosphorus concentrations in the undiluted dairy wastewater influent stream using sodium nitrate (Thermo Fischer, Pittsburgh, PA) and potassium phosphate (Thermo Fischer, Pittsburgh, PA). The final organic carbon content of the wastewater dilutions was set to 1200, 600, and 300 mg L^-1^ of total organic carbon. The N:P ratio was balanced weekly to the same 155 mg:12 mg ratio to accommodate for the uptake of nutrients by the biofilm. This experiment was conducted utilizing a semi-batch system, with a hydraulic retention time (HRT) of 7 days.

A water bath (VWR) with ¼ in. dia. (0.635 cm dia.) stainless steel tubing was used to maintain the water temperature of the reactors at 7, 17, or 27 ° C (±0.5 ° C). This range of temperatures was chosen as a representative range of seasonal water temperatures in Northern Utah [21]. Constant light was provided from eight 40 W fluorescent lamps that provided a total of 200 μmol photons m^-2^ s^-1^ of continuous photosynthetically active radiation to the upper surface of the RABR systems. Two grams of centrifuged wet weight of adapted inoculum were added to the cotton rope growth substratum upon initiating rotation of the reactors.

### Biomass determination and quantification

Biomass was harvested from the rope substrata weekly by mechanical scraping and lyophilized for biomass determination, ash free dry weight (AFDW) measurements, and chemical composition. AFDW calculation was determined using lyophilized biomass at 550 ° C. Biomass productivity was calculated using the AFDW of the biofilm divided by the areal footprint of the reactor (0.0338 m^2^). Growth rates were calculated, and an Arrhenius plot of the data was used to obtain the temperature correction coefficient. ANOVA calculations were based on using biomass productivity as the dependent variable. Total theoretical productivity for the reactor was also calculated using measured growth rates. The Arrhenius equation was used to model the effect of temperature on the biofilm growth rate.

### Statistical analysis

Triplicate RABR trials for each combination of organic carbon concentration and temperature were conducted for statistical analysis. Each temperature was evaluated by testing three levels of organic carbon in triplicate for a total of nine reactors at each temperature and each organic carbon level. The total number of RABRs was 27, three for each combination of organic loading and temperature. Error bars show 95% confidence intervals from the mean in all figures. ANOVA was performed using triplicate data points in each trial.

## Results and discussion

### ANOVA

In this study, biofilm productivity was the quantitative outcome, and temperature and organic loading were the explanatory variables. The advantage of performing ANOVA is that it not only provides a means to see how both of the independent variables, temperature and concentration of organic carbon, impact the dependent variable, biomass productivity, but also how the interaction of the two independent variables impacts the dependent variable [22].

Results of ANOVA can be seen in Table 2. With *p*-values of less than 0.005, both increasing temperature and increasing organic carbon concentration were correlated with an increase in biofilm productivity. However the interaction of temperature and organic loading did not contribute to a statistically significant increase in productivity (*p*-value 0.8871).

**Table 2:** Summary of the Analysis of Variance (ANOVA) results for the effect of temperature and organic carbon concentration on productivity of RABR based algae biofilm

Source	Sum of squared deviations	Degrees of freedom	Mean square	F-Statistic	*P*-Value
Temperature	14.12	2	7.06	8.87	*0.0021*
TOC Concentration	30.2	2	15.1	18.98	*<0.0001*
Interaction	0.88	4	0.22	0.28	*0.8871*
Error	14.32	18	0.8		
Total	59.52	26			

### Temperature effects on system productivity

Because ANOVA results indicate that temperature was a contributing factor to biomass productivity, biofilm productivity rates at the three specified temperatures were applied to the Arrhenius equation in order to obtain the activation energy (*E*_*a*_). The equation used was

1 $$ K=A*{e}^{\frac{-{E}_a}{RT}} $$

where *K* is the biomass productivity, *E*_*a*_ is the activation energy of the reaction, and *R* is the universal gas constant (8.314 J K^-1^ mol-1). Equation 1 was then linearized by taking the natural log of both sides.

2 $$ lnK=\frac{-{E}_a}{RT}+ ln\;A $$

**Fig. 2:**
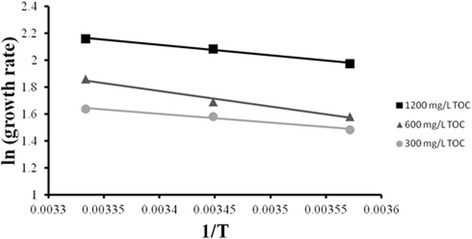
Arrhenius plot of RABR productivity (g · m^-2^ day^-1^) as a function of temperature (K). The slope of the best fit line for each concentration represents $\frac{-{E}_a}{R}$ where *R* = 8.314 *J K* ^− 1^ *mol* ^− 1^ and *E*_*a*_ was calculated (Table 3)

The slope of the line formed after plotting *lnK* vs. - $\frac{1}{T}$ provides the activation energy (*E*_*a*_) (Fig. 2). Using the Van’t Hoff-Arrhenius equation, it was possible to apply the activation energy to derive the equation

3 $$ \frac{K_2}{K_1}={\theta}^{T_2-{T}_1} $$

in order to find the temperature correction coefficient; theta (*Θ*) [23]. The activation energies and temperature correction coefficients are reported in Table 3, and were observed to be consistent with values seen in other biological systems [24–26].

**Table 3:** Temperature correction coefficients, activation energies, and constants of biofilm productivity and nutrient uptake at three levels of organic loading (TOC)

Level of TOC (mg/L)	Symbol	1200	600	300
Biomass Productivity (g m^-2^ day^-1^)	K	8.69	6.44	5.15
Activation Energy (J K^-1^ mol^-1^)	E_a_	6473	9739	5440
Temperature Correction Coefficient (unitless)	Θ	1.0096	1.0145	1.0081
Nitrogen Uptake Rate (mg m^-2^ day^-1^)	K_N_	1.22	0.91	0.723
Nitrogen Correction Coefficient (unitless)	Θ_N_	1.0098	1.0151	1.0078
Phosphorus Uptake Rate (mg m^-2^ day^-1^)	K_P_	0.17	0.12	0.1
Phosphorus Correction Coefficient (unitless)	Θ_P_	1.0101	1.0149	1.0116

### Organic carbon concentration effects on system productivity

**Fig. 3:**
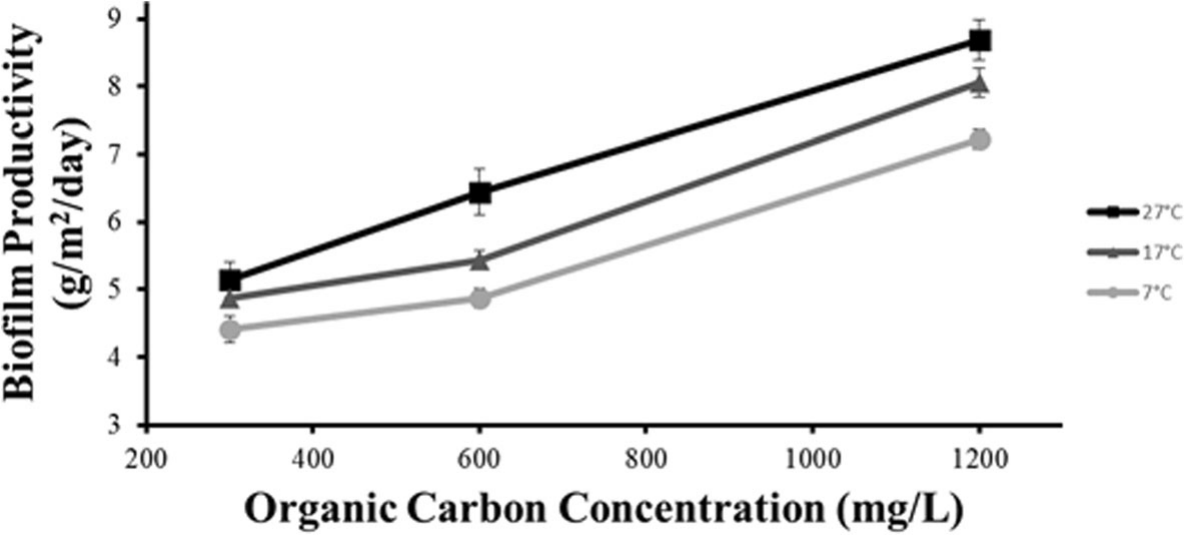
Areal biofilm productivity as a function of organic carbon concentration at three different temperatures. Error bars represent ± 95% confidence interval. *n* = 3 for each data point. *n* = 27 for entire system

ANOVA results indicated that increasing the concentration of organic carbon had a positive correlation with biofilm productivity, and the effect of organic carbon concentration on biofilm productivity is presented in Fig. 3. Application of an algae-based biofilm system for nutrient uptake in dairy wastewater requires that the biofilm be capable of growth in the presence of high levels of organic carbon. The positive correlation between productivity and organic carbon concentration indicates that a biofilm system could be used to remove nutrients from a waste stream with elevated levels of organic carbon. This positive correlation could be due to a symbiotic effect of natural bacteria providing carbon dioxide for phototrophic growth. In a dairy waste stream of similar elemental composition to that of the Caine Dairy, which produces 4000 gal day^-1^, the theoretical yield for AFDW biomass is 9.5 kg day^-1^ of algae-based biofilm.

### Effect of interaction of temperature and organic carbon concentration on system productivity

Because ANOVA calculations indicate that temperature and organic loading did not interact to contribute to growth rate, an Arrhenius linearization of the data is advantageous. Application of biomass productivity (*K*) to Eq. 2 yields values of *E*_*a*_. Both *K* and *E*_*a*_ values are reported in Table 3. These values allow productivity to be compared directly to temperature, creating a predictive system that can be used to estimate biofilm productivity at a given temperature.

At a known concentration of organic carbon, the temperature correction coefficient (*Θ*), derived from Eq. 3, allows for prediction of biofilm productivity at any temperature within the range evaluated, i.e. 7–27 ° C. At a given water temperature and concentration of organic carbon, the *Θ*-values from Table 3 can be applied to the equation

4 $$ {K}_{prediction}=5.152*{\theta}^{T_{water}-280} $$

to predict algal biofilm productivity. A larger *Θ* value indicates a more significant increase in growth rate as temperature increases.

Using molar ratios of the algae-based biofilm, it was also possible to derive a temperature correction coefficient from the biofilm productivity values in order to predict nitrogen and phosphorus uptake by the system. Rates of nitrogen and phosphorus uptake are reported in Table 3 as *K*_*N*_ and *K*_*P*_ respectively, and the temperature correction coefficients for nitrogen and phosphorus, *Θ*_*N*_ and *Θ*_*P*_, are also reported in Table 3. At a known water temperature and concentration of organic carbon, the *Θ*_*N*_-values from Table 3 can be applied to the equation

5 $$ {K}_{N, prediction}=0.723*{\theta_N}^{T_{water}-280} $$

to predict rate of nitrogen uptake (mg m^-2^ day^-1^), and the *Θ*_*P*_-values from Table 3 can be applied to the equation

6 $$ {K}_{P,\  prediction}=0.098*{\theta_P}^{T_{water}-280} $$

to predict rate of phosphorus uptake (mg m^-2^ day^-1^) by the biofilm.

### RABR system productivity

**Fig. 4:**
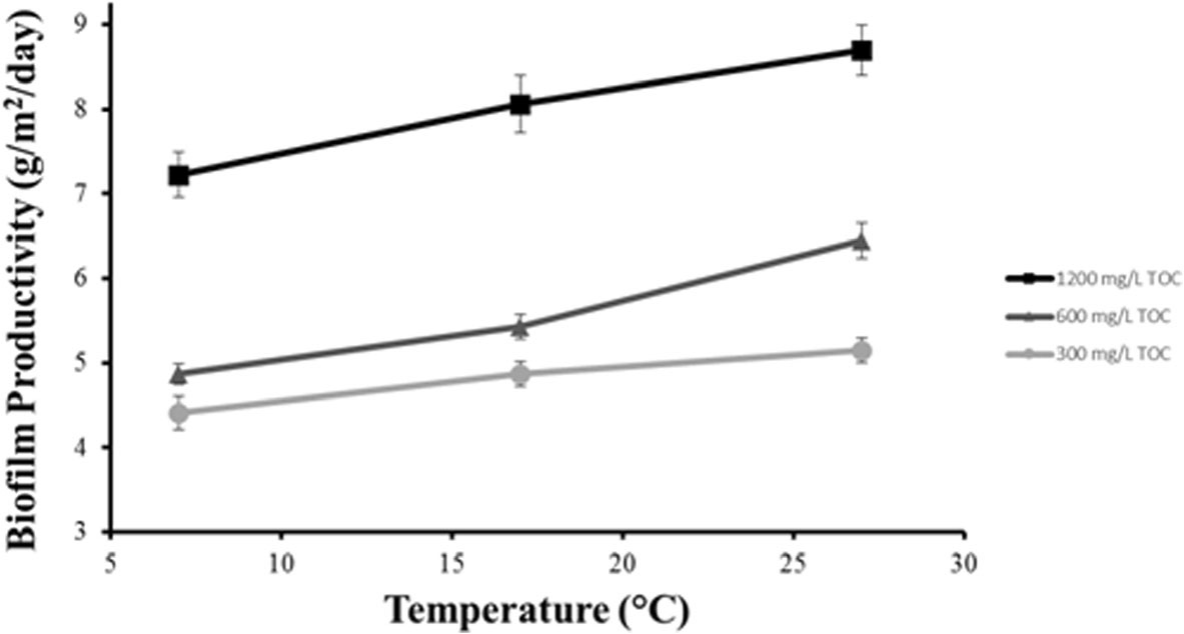
Areal biofilm productivity (AFDW) as a function of temperature at three levels of organic loading. Error bars represent ± 95% confidence interval. *n* = 3 for each data point. *n* = 27 for entire system

After harvesting the produced biofilm, the productivity of the system was calculated. Temperature was made to be a limiting factor for the growth of algae by providing nutrients in excess under constant light [27, 28]. Controlling temperature as the limiting factor allows for the evaluation of the effect of temperature on biofilm productivity, as shown in Fig. 4.

## Conclusions

Results of this research are the first in the refereed literature that the authors are aware of that determined growth rates of algae-based biofilm on dairy wastewater at different temperatures for different strengths of the wastewater. Equations 4, 5 and 6 can be used for a waste stream with known organic carbon concentration and water temperature to predict biofilm productivity and nutrient uptake; where *Θ* is the value taken from Table 3 and *T*_*water*_ is water temperature in degrees Kelvin from 280 to 300. The relationships among temperature, productivity, and TOC developed in this study can be applied to the design of dairy wastewater remediation systems and algae-based biomass production systems using biofilm reactors.
